# A Comparison between Finite Element Model (FEM) Simulation and an Integrated Artificial Neural Network (ANN)-Particle Swarm Optimization (PSO) Approach to Forecast Performances of Micro Electro Discharge Machining (Micro-EDM) Drilling

**DOI:** 10.3390/mi12060667

**Published:** 2021-06-07

**Authors:** Mariangela Quarto, Gianluca D’Urso, Claudio Giardini, Giancarlo Maccarini, Mattia Carminati

**Affiliations:** Department of Management, Information and Production Engineering, University of Bergamo, Via Pasubio 7/b, 24044 Dalmine, Italy; gianluca.durso@unibg.it (G.D.); claudio.giardini@unibg.it (C.G.); giancarlo.maccarini@unibg.it (G.M.); mattia.carminati@unibg.it (M.C.)

**Keywords:** forecast, micro-EDM, FEM, ANN, PSO

## Abstract

Artificial Neural Network (ANN), together with a Particle Swarm Optimization (PSO) and Finite Element Model (FEM), was used to forecast the process performances for the Micro Electrical Discharge Machining (micro-EDM) drilling process. The integrated ANN-PSO methodology has a double direction functionality, responding to different industrial needs. It allows to optimize the process parameters as a function of the required performances and, at the same time, it allows to forecast the process performances fixing the process parameters. The functionality is strictly related to the input and/or output fixed in the model. The FEM model was based on the capacity of modeling the removal process through the mesh element deletion, simulating electrical discharges through a proper heat-flux. This paper compares these prevision models, relating the expected results with the experimental data. In general, the results show that the integrated ANN-PSO methodology is more accurate in the performance previsions. Furthermore, the ANN-PSO model is faster and easier to apply, but it requires a large amount of historical data for the ANN training. On the contrary, the FEM is more complex to set up, since many physical and thermal characteristics of the materials are necessary, and a great deal of time is required for a single simulation.

## 1. Introduction

Micro Electro Discharge Machining (micro-EDM) is a non-conventional process able to remove material from the workpiece by means of the thermal energy generated by rapid electric sparks, occurring between electrode and workpiece in a dielectric medium that separates the two elements. It is widely used for making micro features in difficult-to-cut materials. In particular, it is successfully applied for executing micro-holes in several fields such as automotive (e.g., the nozzle of gasoline and diesel injectors), aerospace (e.g., cooling holes of turbine blades), textile (e.g., for spinnerets tools), micro-mechanicals (e.g., watch components), and medical (e.g., micro-fluidic devices). The micro-EDM is a contactless material removal process in which the sparks cause the melting and the vaporizing of the material since they develop an intense amount of heat which is transferred to the workpiece surface in a very focused area. In this way, the workpiece temperature locally increases exceeding the melting temperature and, thanks to the vaporization phenomenon and the dielectric flush, the material is removed from the workpiece surface.

The phenomena occurring during the electrical discharge process are still the object of several studies and different models for the prediction of actual removing mechanism can be found in literature and are used as a basis for predicting the main process performances. In particular, numerical models have been developed for simulating the spark erosion process, while analytical models, developed combining different optimization algorithms, were used for forecasting the process performances and optimizing the process parameters combinations. Among all the developed methods, FEM is noteworthy. In [1] the temperature distribution and the thermal stress caused on the workpiece due to the applied heat flux during a single spark discharge were investigated. The development of compressive and tensile stresses in a thin layer around the discharge area, which could damage the surface, was observed. The FEM was used to evaluate the crater dimensions, the material removal rate (*MRR*), the residual stresses, and the phase transformations on the cathode after the occurrence of sparks; the temperature dependence on material properties, the inclusion of latent melt heat, the Gaussian distribution of heat flux, and the spark radius dependence on peak current and discharge width were taken into account [2,3]. The effect of different parameters on the temperature distribution of the cathode was analyzed, showing that the temperature distribution and the crater dimension are directly proportional to the values of current, voltage and duty cycle. However, the crater dimension increases until a certain value of discharge duration while the crater dimension decreases for further increments in discharge duration [4].

To make the FEM closer to the real EDM process, it is important to consider, for a single-spark model, the variability as a function of the operating parameters. For example, in [5] the authors took into account the variable nature of the energy fraction and the plasma flushing efficiency as a function of both discharge current and pulse on-time. In this work, the plasma flushing efficiency was used as a correction term, applied following the simulation, to calculate the crater dimension.

Regarding the analytical model, due to a large number of involved variables, the complexity of the process and the fact that the dimensional precision of the part improves as the process performance (*MRR*) gets worse, it is difficult to accurately predict the process performances and the part quality as a function of the process parameters. Thus, process optimization becomes a very hard task. For this reason, techniques such as regression analysis, artificial neural network (ANN), multi-objective optimization techniques and other optimization algorithms like a genetic algorithm (GA) and surface response (SR) have received great attention from researchers over the past 10 years. In particular, the ANN remains one of the most applied techniques for process modelling, but, once trained, it is used coupled with optimization algorithms such as genetic algorithm, resident advisor algorithm, or response surface. For example, an integrated method using artificial neural network (ANN) and genetic algorithm (GA) was used for analyzing the material removal rate (*MRR*) and for optimizing the process parameters, showing errors within acceptable limits and determining optimum process parameters for the desired output value through the GA [6].

In [7] an ANN was developed and used to predict the surface roughness in wire-EDM (WEDM) of Cr-Mo-V special steel, applied in the automotive industry. The neural network training was performed with experimental results obtained using a Taguchi method. The mathematical relation between the workpiece surface roughness and WEDM cutting parameters was established by multiple regression analysis methods. Predicted values of surface roughness by back-propagation (BPN) and general regression neural networks (GRNN) were compared with the experimental values and their closeness was analyzed, showing that values in the BPN network with two hidden layers are closer to the experimental results than the GRNN network and multiple regression values. The artificial neural network (ANN) was also used in the development of a predictive model of the material removal rate (*MRR*) in EDM using an input-output pattern of raw data coming from process experiments for copper-electrode and steel-workpiece [8].

Based on the literature review, the focus of the optimization process can be dedicated to the study of specific workpiece material, the investigation of a single process performance parameter, or the analysis of a specific EDM configuration.

In several works, the study was focus on the specific workpiece material. For example, a general regression neural network approach on AISI D2 tool steel was applied in [9]. In 2018 a multi-objective optimization, using an integration between the ANN and the non-dominated sorting genetic algorithm II (NSGA-II), was performed on A2 steel [10]. AISI 304 was the material selected for developing a predictive model using signal-to-noise analysis (S/N), response surface methodology (RSM) and ANN in [11]. 17-4PH and AISI 1020 were the materials selected for the prediction of EDM process parameters using ANN and other optimization algorithms in [12,13]. All the solutions reported in these works show that, whatever optimization algorithm is applied to the ANN, it is possible to obtain effective optimization and predictive techniques. In general, these approaches allow forecasting results with an accuracy between 90% and 95%.

In the second category of researches, for example, only the *MRR* was involved in the prediction process of several works that analyze different types of electrodes and workpiece [14,15,16]. In other works, authors focused their attention on the evaluation of surface finishing, assessing the ANN method by investigating its accuracy in predicting the mean surface roughness of the EDM surface. The mean surface roughness predicted from multi-layer feed-forward neural network resulted to be very close to experimental results [17]. The study shows that the alternative ANN approach can be used to successfully predict the microcracking of tungsten carbide during the EDM process. An artificial neural network coupled with the Taguchi approach was applied for optimization and prediction of surface roughness, giving a good agreement between experimental results and predicted results. Furthermore, the study shows that high discharge energy causes surface defects such as cracks, craters, thick recast layer, micropores, pinholes, residual stresses, and debris [18,19]. For example, in [20], the attention is focused on the prediction and comparison of machining performances during wire EDM (WEDM) of Al7075-TiB2 through an ANN model. In all these cases, the models could be considered as characterized by some limitations since they have validity only for wire configuration and it is difficult to modify to expand their applicability to drilling, sinking or milling configurations.

The present study compares new approaches for predicting and optimizing performances and process parameters for the micro-EDM drilling process. In the numerical approach, a FEM model, developed by the authors in previous work [21], was considered for simulating the material removal process by means of a damage routine. This latter model deletes the mesh elements that reach the melting temperature of the material due to the flow of heat transmitted through continuous electrical discharges. For the analytical model, an approach that integrates the Artificial Neural Network (ANN) with the Particle Swarm Optimization algorithm (PSO) was developed and validated. The prediction accuracy of both the forecast methodologies was compared based on main process indicators, such as the Material Removal Rate (*MRR*), machining time, and dimensional deviation.

## 2. Prevision Models

In this section, the developed prediction models were described. In particular, the first numerical method uses a commercial Deform 2D code specific for the simulation of thermo-mechanical forming processes setting up an axisymmetric 2D model; a single step simulation was performed, while an external flux was imposed on the model. For the simulation of a non-conventional process, representing a new possible application, the EDM process can be simulated on its macro aspects. The second method (analytical) is based on the development of an integrated approach, considering the Artificial Neural Network (ANN) combined with the Particle Swarm Optimization (PSO).

### 2.1. FEM Model

The FEM model was based on the capacity of the code to simulate the removal or the separation of the material through the mesh elements detection. Its development and set-up were described in detail in the authors’ previous work [21]. The electrical discharges were simulated by means of suitable heat flux and the material melting temperature was set as a trigger for the element deletion. For developing the FEM code, first of all, the single discharge cycle simulation was set and tested for verifying the good predictive ability. The simulation of the material removal process was realized using a specific external routine based on the deletion of all those elements that reach a specific target temperature, corresponding to the melting temperature of the workpiece material. To assess the effectiveness of the model, it was matched with the models already presented in the literature and both simulations and results were compared.

To simulate a complete process, it was necessary to consider the continuous cycle of the electrical discharges occurring between the electrode and the workpiece. This was modelled by assuming a heat flux corresponding to the thermal energy generated by the average number of discharges occurring in the time unit and randomly distributed on the electrode surface. Beyond the workpiece and the electrode objects, a third symbolic element representing the dielectric was added. This solution was used to allow the heat transfer by conduction because of the absence of contact between the workpiece and electrode. The heat exchange was modelled pushing a constant heat flux on the electrode surface, using a simulated dielectric as a conductive medium and evaluating the fraction transferred to the workpiece and the achieved temperature distribution.

A significant aspect of the modelling was the definition of the properties and the behavior of the material selected to simulate the dielectric medium; a thermal expansion coefficient equal to zero was assumed to keep the volume of the object constant, in order to not affect the shape during the removal process.

The dielectric medium has an important function for the thermal conduction; in fact, in the real EDM process, this medium is characterized by both electrical insulation property (to allow the generation of potential difference) and thermal insulation property (to shield the electrode from the thermal shock and to cool it). The thermal conductivity and the volumetric thermal capacity were set to reproduce the typical properties of hydrocarbon oil.

To simulate the process as truthfully as possible, it was necessary to guarantee a constant distance between the electrode and the workpiece. In the real process, the gap control is performed by a closed-loop system managing the process parameters, such as current, voltage and tool axial stroke. This solution was not available in the simulation; therefore, a constant speed was applied to the electrode. In the contact area between the elements, the heat exchange coefficient was imposed equal to 1000 W/m^2^K, as suggested by the literature [22]. All the workpiece surfaces were considered adiabatic, except for the area in which the heat flux is applied. On the electrode surfaces a heat exchange boundary condition was applied with the external environment, characterized by a temperature equal to 20 °C. The convective exchange coefficient was set equal to 0.02 W/mm K.

An electrode having a tubular geometry was modelled. During these tests, it was observed that the electrode temperature, after a preliminary stage, reached a steady-state condition for the entire machining cycle. In this way, the dielectric was sufficiently hot to guarantee the continuous melting of the workpiece and the moving forward of the electrode itself. The dielectric helps the electrode cooling. Since it was not possible to simulate the continuous recycling of the medium in the working area to allow the heat removal, it was necessary to impose an increased thermal exchange on the top of the dielectric element. In this way, a dielectric flux inside the electrode and the removal of part of the generated heat was simulated, maintaining stable operating temperatures and allowing the process to proceed stably.

To guarantee a realistic thermal effect, another modelling solution was added: the thermal exchange between the electrode and the dielectric medium was hindered near to the end of the electrode (in the inner part, for some hundred microns), applying a small gap to avoid the touch between the two elements. Thanks to this approach, the flow can be directed mainly towards the electrode, while the dielectric continues to act as a cooler in the upper part of the electrode, from where the heat is removed.

A specific damage model was set up to eliminate the elements of the workpiece during the simulation. This model is based on a parameter that represents the value of a physic entity, such as the temperature or the strength. This value can be calculated in each simulated step and for all the mesh elements. A threshold can be set and, when an element reaches this value, it is canceled in the following step. In this case, the workpiece melting temperature was assumed as a threshold condition.

In general, the FEM model was based on the imposition of a heat flux estimation as reported in Equation (1).

(1) $$Q=F_{c}VI/\pi r_{c}^{2}$$

where *F_c_* is the fraction of energy transferred to the cathode, *V* and *I* represent voltage and peak of current of micro-EDM machining, respectively, and *r_c_* is the radius of the heat source. The model resulted to be able to reproduce with accuracy the geometry and it resulted to be comparable to the real process in terms of Material Removal Rate (*MRR*) and machining time (*t*) [21].

### 2.2. ANN-PSO Model

#### 2.2.1. ANN Design

The ANN-PSO model is a 2-step optimization methodology characterized by a bidirectional functionality. The neural network was designed and trained based on the historical data about the micro-EDM drilling performed under several conditions in terms of process, workpiece and electrode characteristics (material and electrode diameter). The second step of the model involved the PSO algorithm in order to use the trained ANN for identifying optimal process parameters under different multi-objective functions and by introducing several constraints in the solution space definition. The main advantage of this model is the possibility to effectively work when constraints are fixed not only for the ANN inputs (process parameters and/or workpiece and electrode characteristics) but also for the ANN outputs (process performances), respecting the imposed multi-objective function that aims to maximize the Material Removal Rate (*MRR*) and minimize the machining time and the dimensional deviation. In this way, the model can respond to the industrial scenario externally imposing some input and/or output at the same time, based on the operator’s decision of maximizing and optimizing the productivity and the technical specifications defined by the design.

The ANN was built considering six inputs (*IN*) and three outputs nodes (*ON*). Specifically, the input nodes are represented by the peak current (*I*), voltage (*V*), frequency (*F*), electrode diameter (ϕ), workpiece material (*WP*), and electrode material (*El*). The output nodes are represented by the material removal rate (*MRR*), the machining time (*t*), and the dimensional deviation (*DD*). For the hidden layer, based on the literature review, a single hidden layer may be sufficient, while the right number of Hidden Nodes (HN) is the most challenging and interesting aspect in the definition of an optimized ANN structure. Creating an ANN with too few or too many HN can generate underfitting or overfitting situations. For selecting the eligible number of HN in the single hidden layer, a range in which the number of neurons varies was considered taking into account some heuristic methods to define the upper and the lower limit for the HN as a function of *IN* and *ON*. Specifically, according to the literature [23,24], the lower bound (*MTI*) was defined as reported in Equation (2).

(2) $$MTI=\frac{IN+ON}{2}$$

The upper bound of HN was defined as the maximum values obtained between the Kudrycky [24] (Equation (3)), Kolmogorov [25] (Equation (4)), and Lippman [26] (Equation (5)) equations.

(3) KUD=3×ON

(4) KOL=2×IN+1

(5) LIP=ON×(IN+1)

**Figure 1:**
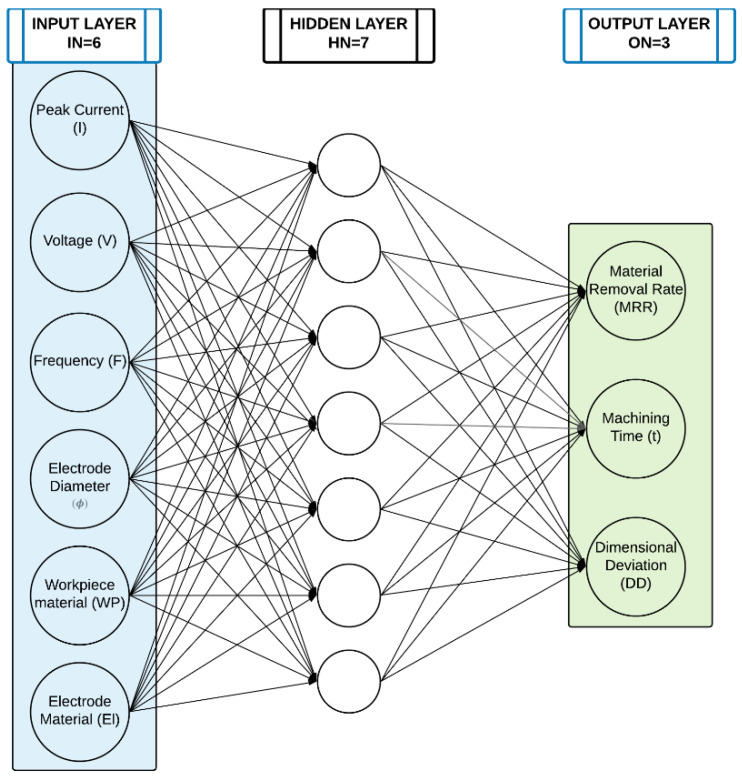
ANN structure.

Through a MATLAB code, the ANN performances were tested varying the HN in the range 5 (*MTI*)–21 (*LIP*). For identifying the best ANN configuration, the Coefficient of Variation (*CV*) was selected for the error evaluation. CV is a statistical indicator independent from the distribution giving a meaningful evaluation. It was defined as the ratio between standard deviation and the average value of Root Mean Square Error ($\sigma_{RMSE}{}_{i}$ and $\mu_{RMSE_{i}}$) of each output i. Fixing the number of hidden nodes, the sum of *CV_i_* (where *i* identifies the correlated output) was estimated and, as optimal configuration, the one characterized by the lower level of prevision error was selected ($\min CV_{i}$). Figure 1 shows the optimal configuration and details about selected input and output nodes.

**Figure 2:**
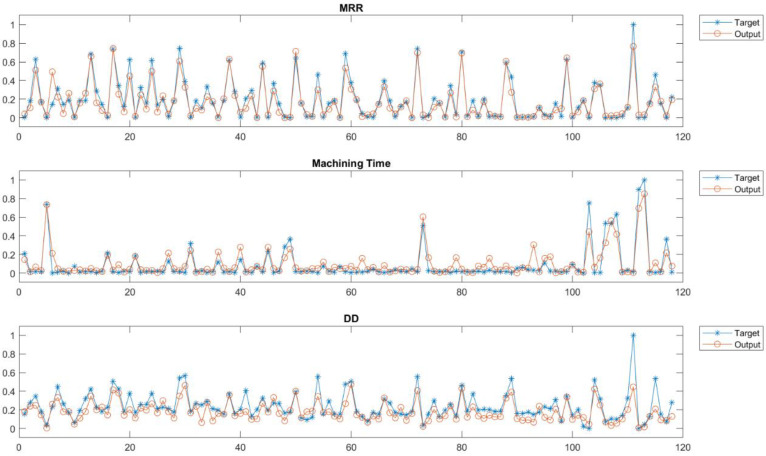
Prediction test data and actual values of Dimensional Deviation (Data close to 0 has a magnitude order equal to 10^−6^).

All input and output nodes are normalized between 0 and 1 to avoid effects due to the large differences in the actual values of the variables. Once the ANN structure was defined, the network required training, validation, and testing. The dataset involved in the definition of the ANN was collected on a Sarix SX-200 (Sarix SA, Sant’Antonino, Switzerland) micro-EDM machining. 70% of the dataset was used for the training, 15% for the test, and 15% for the validation. Figure 2 shows the predicted data (Output) through the ANN and the actual values (Target). For all analyzed output (*MRR*; Machining Time and *DD*), it is possible to note that predicted data well fit the actual values of the outputs.

#### 2.2.2. PSO Algorithm

Such a trained ANN is able to reliably predict the process performance once the working conditions were defined (fixing the input values), but it is not able to optimize the process since an objective function is not defined. For this reason, an optimization algorithm was introduced. Among the various developed algorithms, Particle Swarm Optimization was chosen for this study. In PSO the potential solutions fly, simulating a flock of birds, through the problem space by following the current optimum particles which are those that better satisfy the imposed objective function.

**Figure 3:**
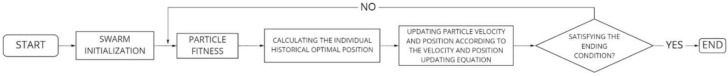
General functionalities of the PSO algorithm.

In this work, the possible values for the ANN represent the problem space of PSO in order to find the input values that minimize the objective function. The general functionalities of PSO are reported in the flowchart in Figure 3.

In this specific case, a multi-objective function was selected as an objective function since the outputs considered in the present research (*MRR*, Machining Time, and *DD*) are conflicting because it is important to increase the *MRR*, while Machining Time and *DD* should be minimized. The multi-objective function is reported in Equation (6).

(6) minf(I, V,F, ∅,WP, El)=t+DD−MRR

where f(I, V,F, ∅,WP, El) is an unknown function substituted by the trained ANN.

#### 2.2.3. Model Validation

**Figure 4:**
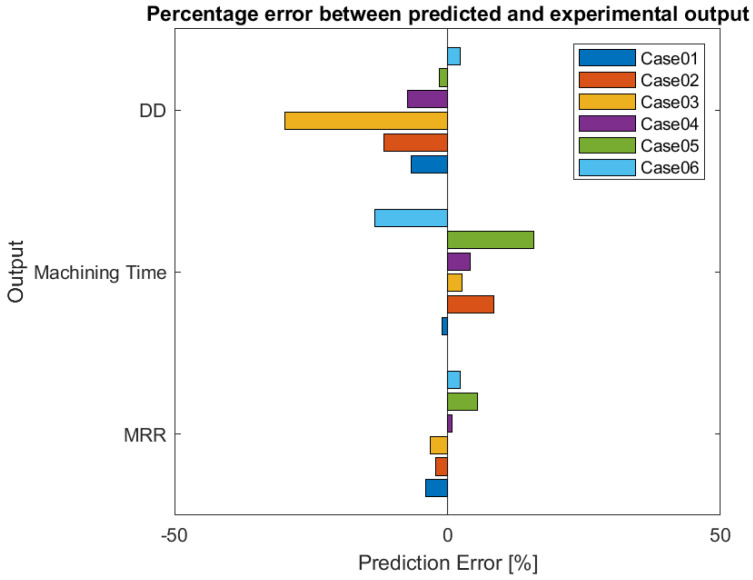
Percentage error between predicted and experimental process performances.

The main characteristic of this 2-step model is the possibility of forcing into the developed Matlab code both input and/or outputs. For the model validation, it was selected to force into the model the characteristics of the workpiece and the electrode for optimizing the process parameters, minimizing the objective function. In Table 1, the forced input and the optimization results in terms of process paraments are reported. The outputs obtained through the integrated ANN-PSO model were compared with the experimental results to define the forecast precision. The experimental tests were performed on plates with a thickness equal to 3 mm and the process parameters set up for the drilling machining were defined through the optimization process performed by means of the developed model for verifying the system reliability. The forecast percentage error achieved by the model can be observed in Figure 4, where it is possible to observe that the deviation between the forecast and experimental performances is less than 30%.

## 3. Experimental Procedure

In the present work, one of the authors’ aims was to underline a possible different accuracy in the prevision capacity of the different methodologies and to investigate if one method could be defined as better than the other. To do that, some experimental cases were taken into account. In particular, some drilling tests were performed using a Sarix SX-200 micro-EDM machine (Sarix SA, Sant’Antonino, Switzerland). A stainless steel AISI304 having a thickness equal to 3 mm was used as a workpiece and two different materials and diameters were considered for the electrode. Specifically, 150 µm and 300 µm were selected as external diameters for both tungsten carbide and brass tubular electrodes. Hydrocarbon oil was used as a dielectric fluid. Table 2 reports the properties of both workpiece and electrode materials. Differences in the electrode gave rise to differences in terms of heat flux and machining speed, decisive factors for the simulation models with which the results were compared.

Three different sets of process conditions (Table 3) were selected for the tests varying the main process parameters (peak current, voltage, and frequency). The selection of variable process parameters and the corresponding levels was based on the input parameters taken into account in the developed simulative models, considering the factors necessary for the definition of the heat flux in the FEM model. For the experimental tests, two runs for each combination were performed.

Based on the prevision models and their outputs, during the experimental tests, the machining time was recorded and the *MRR* and the geometrical deviation of the diameter from the nominal value were calculated as reported in Equations (7) and (8), respectively.

(7) $$MRR=\frac{VMR}{t}$$

(8) $$DD=D_{hole}-D_{el}$$

where the hole diameters ($D_{hole}$) were measured by means of an optical microscope, while $D_{el}$ refers to the nominal diameter of the hole which matches with the electrode diameter.

## 4. Comparison of FEM and ANN-PSO Model with Experimental Results

The experimental results were compared with the predicted data obtained through both the FEM and ANN-PSO models. For the integrated ANN-PSO model, the process parameters used for the experiments were fixed into the PSO step to obtain the optimal outputs. In the FEM model, the process parameters were used to define the heat flux identifying three intensity levels (low, medium, and high). Specifically, for each level of parameters set, a different value of heat flux was estimated considering the process parameters applied in the experimental tests, as reported in Section 2.1. In this way, the heat flux set into the FEM model was varied on three levels indicated as low, medium, and high, as reported in Table 4.

Once the experiments and the simulative model were run, the data related to the output indicators were collected and the deviation between the experimental and the simulative results were estimated as a percentage for identifying the differences and defining the different behavior of the models.

**Figure 5:**
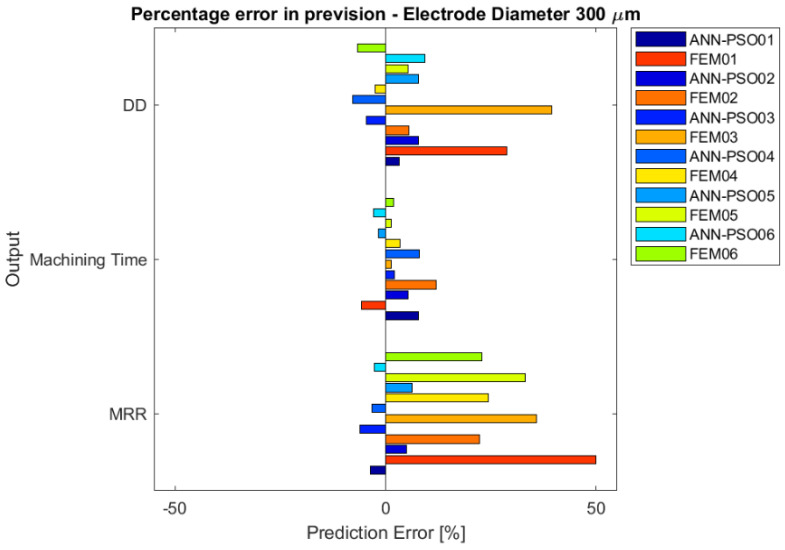
Bar chart of percentage error between simulation models and experimental tests, for drilling process performed by means of an electrode with a diameter equal to 300 µm.

**Figure 6:**
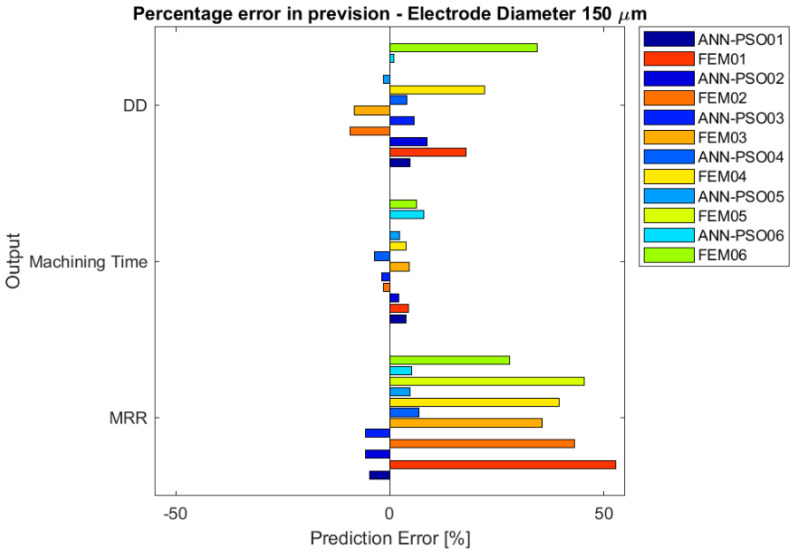
Bar chart of percentage error between simulation models and experimental tests, for drilling process performed by means of the electrode with a diameter equal to 150 µm.

The percentage errors are summarized in Figure 5 and Figure 6 as a function of the electrode diameter. Both models predicted the results with certain stability; regardless of the tested conditions, the results are consistent with each other, showing repeatability and consistency in the previsions. Considering the different electrode diameters, the simulation appears to have similar behavior. In particular, it is possible to state that the FEM model makes greater forecasting errors than the integrated ANN-PSO model in estimating *MRR* and Dimensional Deviation, showing a percentage of error up to 50% (especially overestimating). The machining time resulted to be the better simulated indicator in both the developed models.

Considering the general evidence of the error estimation, it is clear that the ANN-PSO simulation fits better with the experimental results, maintaining a low percentage of error for all the indicators in all the tested situations.

## 5. Conclusions

This work compared two different simulation techniques for forecasting the performance indicators of the micro-EDM process. In particular, in this case, three indicators were taken into account even though both models could be modified by introducing more or different indicators.

The FEM model was developed by the authors in a previous work where the model definition and the validation are described in detail. The integrated ANN-PSO model was introduced presenting the ANN configuration, the PSO algorithm for optimization, and the validation.

A comparison between the application of these two models was performed considering six different drilling tests, where two different types of electrode materials and geometry were taken into account.

For each type of electrode, three machining conditions were tested evaluating different levels of machining (low, medium, and high).

The experimental data were compared with the data provided by the two simulation methods and the percentage deviation between predictive approaches and experiments was calculated for evaluating the reliability and precision of the forecast. In this way, it was possible to define if one of the models is more accurate than the other and which is the error that we can expect from the application of these methodologies.

In general, the comparison shows a low percentage of error for the integrated ANN-PSO method for the dimensional deviation and the material removal rate, while the machining time results to be the indicators better predicted by both models. Despite this consideration, it is important to remark that to build an effective trained ANN, a great amount of data is necessary. On the other side, running a FEM simulation requires a lot of time for data elaboration and for computing the information.

## Figures and Tables

**Table 1:** Forced inputs and optimized process parameters.

Input	Case	Imposed Input	Predicted Input	Case	Imposed Input	Predicted Input
ϕ	Case 01	300 μm	60	Case 02	300 μm	56
*WP*		WC	153		WC	110
*El*		Brass	130		WC	100
ϕ	Case 03	150 μm	40	Case 04	100 μm	100
*WP*		AISI 316L	95		AISI 316L	120
*El*		WC	120		WC	150
ϕ	Case 05	300 μm	140	Case 06	300	86
*WP*		Al	130		Al	115
*El*		WC	90		Brass	100

**Table 2:** Micro-EDM main process parameters.

Physical Property	AISI 304	Tungsten Carbide	Brass
Density (g/cm^3^)	8.00	14.80	8.47
Melting Temperature (K)	1673.15–1728.15	3140.15	1178.15–1203.15
Electrical resistivity (Ω·cm)	72 × 10^−6^	20 × 10^−6^	6.63 × 10^−6^
Thermal conductivity (W/mK)	16.2	70	121
Specific heat (J/g · K)	0.50	0.30	0.38

**Table 3:** Micro-EDM main process parameters.

Electrode Material	Electrode Diameter	Level	Peak Current (*I*-Index)	Voltage (*V*-V)	Frequency (*F*-kHz)
Tungsten Carbide	300 µm	Low	23	110	110
		Medium	37	115	115
		High	50	120	120
	150 µm	Low	20	70	110
		Medium	40	95	115
		High	60	110	120
Brass	300 µm	Low	40	135	130
		Medium	60	143	135
		High	80	150	140
	150 µm	Low	10	70	120
		Medium	15	95	120
		High	20	110	120

**Table 4:** Parameters setup for the FEM simulation tests.

Electrode Diameter		300 µm	150 µm
Electrode Material	Level	Heat Flux [kW/mm^2^]	Heat Flux [kW/mm^2^]
Tungsten carbide	Low	8.95	19.81
	Medium	15.05	53.76
	High	21.22	93.37
Brass	Low	19.10	9.90
	Medium	30.35	20.10
	High	42.40	31.10
